# Failure of primary ACL repair with dynamic intraligamentary stabilization may result in a high risk of two-stage ACL reconstruction: a case series of ten patients

**DOI:** 10.1186/s40634-022-00519-2

**Published:** 2022-08-17

**Authors:** Riccardo Cristiani, Caroline Mouton, Renaud Siboni, Charles Pioger, Romain Seil

**Affiliations:** 1 Department of Molecular Medicine and Surgery, Stockholm Sports Trauma Research Center, Karolinska Institutet, Stockholm, Sweden; 2 Capio Artro Clinic, FIFA Medical Centre of Excellence, Sophiahemmet Hospital, Valhallavägen 91, 11486 Stockholm, Sweden; 3 Sports Clinic, Centre Hospitalier de Luxembourg-Clinique d’Eich, 78 Rue d’Eich, 1460 Luxembourg, Luxembourg; 4 Sports Medicine and Science, Luxembourg Institute of Research in Orthopaedics, Luxembourg, Luxembourg; 5 Department of Orthopaedic Surgery, Reims Teaching Hospital, Hôpital Maison Blanche, 45 Rue Cognacq-Jay, 51092 Reims, France; 6 Department of Orthopaedic Surgery, Ambroise Paré Hospital, Paris Saclay University, 9, avenue Charles de Gaulle, 92100 Boulogne-Billancourt, France; 7 Orthopaedics, Sports Medicine and Digital Methods, Human Motion, Luxembourg Institute of Health, Strassen, Luxembourg

**Keywords:** Anterior cruciate ligament reconstruction, ACL, ACL repair, Dynamic Intraligamentary Stabilization, Failure, ACL reconstruction, Revision surgery

## Abstract

**Purpose:**

Dynamic Intraligamentary Stabilization (DIS) is a technique for the repair of acute anterior cruciate ligament (ACL) injuries. The purpose of this study was to investigate the potential challenges of ACL reconstruction (ACLR) following failure of DIS.

**Methods:**

A retrospective analysis of patients with failure of primary ACL repair performed with DIS was undertaken. Failure was defined as abnormal knee laxity (positive Lachman and/or pivot shift) and/or severely restricted range of motion. Medical and surgical records were reviewed and preoperative standard anteroposterior and lateral X-rays were assessed.

**Results:**

Between July 2015 and May 2022, 10 patients (3 males, 7 females, median age 28 years, range 18–52 years) with failure of DIS were referred to and surgically treated at a single centre. In four patients, single-stage ACLR was performed following the removal of the tibial monoblock. In six patients, arthrofibrosis and excessive tibial tunnel enlargement following the removal of the monoblock prevented single-stage ACLR. These patients underwent arthroscopic arthrolysis and tibial tunnel bone grafting as a first-stage revision procedure.

**Conclusion:**

In the present case series, single-stage ACLR was performed in only four (40%) of ten patients following failure of ACL repair with DIS. Arthrofibrosis and excessive tibial tunnel enlargement following the removal of the monoblock prevented single-stage ACLR in six (60%) patients. It is important for clinicians to inform patients that, in the event of failure of ACL repair with DIS, they may run a high risk of undergoing two-stage ACLR.

**Level of Evidence:**

Level IV, Case Series.

## Introduction

An anterior cruciate ligament (ACL) tear is a common injury, which can lead to knee instability, interfering with sports and activities of daily living. ACL injuries can be treated both conservatively and surgically. In the event of surgical treatment, anatomical ACL reconstruction (ACLR) is accepted as the gold standard [1, 2]. However, in an attempt to reduce graft morbidity and preserve the proprioceptive function of the native ACL, there has been increasing interest in ACL repair in recent years [3–7]. Dynamic Intraligamentary Stabilization (DIS) has been proposed as an alternative treatment for the repair of acute proximal ACL tears [8]. The technique is based on trans-osseous suture ACL repair with a dynamic internal brace using a supportive spring-screw mechanism, which maintains posterior tibial reduction through range of motion, theoretically reducing cyclic loads on the ACL during the healing phase [9]. The device is commercially available under the name Ligamys© (Mathys AG, Bettlach, Switzerland). Several studies, conducted by the developers, have reported the restoration of knee laxity, excellent clinical outcomes and a return to the pre-injury activity level after DIS [8–12]. Studies conducted by other authors have, however, reported contrasting results. At a one-year follow-up, Meister et al. [13] found a re-tear rate of 15% and a re-operation rate of 35% due to re-tear or arthrofibrosis. Osti et al. [14] found an overall complication rate (including re-rupture or non-healing, repeat arthroscopy due to meniscus tears, cyclops syndrome, restricted range of motion, arthrofibrosis, implant interference) of 57.9% at a one-year follow-up. Moreover, the authors reported persistent anterior side-to-side laxity of ≥ 3 mm in 49.1% of cases. Finally, a recent study based on 57 patients who underwent DIS after an acute proximal ACL rupture revealed a five-year survival rate of 70%, which dropped to 56% for patients active in competitive sports before the injury [15]. Nevertheless, there is a lack of literature evaluating the potential challenges of ACLR following failure of ACL repair with DIS. The purpose of this study was to review the difficulties related to ACLR following failure of DIS in a series of patients referred to our institution. It was hypothesised that, in most cases, single-stage ACLR would be difficult to perform following failure of ACL repair with DIS.

## Materials and methods

The patients included in this case series are part of our ACL clinical pathway. They gave their written informed consent to grant us permission to use their medical data gathered during their treatment. The project has received the approval of the National Ethics Committee for Research (N°201,101/05). Data acquisition and storage were notified to the National Data Protection Committee.

Patients with failure of primary ACL repair with DIS performed elsewhere and referred to Centre Hospitalier de Luxembourg (Luxembourg) were identified. Failure was defined as abnormal knee laxity (positive Lachman and/or pivot shift) and/or severely restricted range of motion. All the revision procedures were performed by the same senior surgeon (RS). Medical and surgical records were reviewed and preoperative standard anteroposterior and lateral X-rays were assessed. The radiological assessment was performed by an orthopaedic knee surgeon (RC). Femoral tunnel position was assessed according to the Bernard and Hertel grid method [16]. The centre of the femoral tunnel aperture was identified on lateral X-rays and its location was reported as percentages of the sagittal width (from posterior to anterior) of the femoral condyle and height (from superior to inferior) of the intercondylar fossa. The tibial tunnel position was assessed using the Staubli and Rauschning method [17]. The centre of the tibial tunnel aperture was identified on lateral X-rays and its location was reported as a percentage of the anteroposterior length of the tibial plateau. In addition, the distance from the tip of the monoblock to the tibial plateau was measured on lateral X-rays and reported in millimetres.

## Results

Between July 2015 and May 2022, 10 patients (3 males and 7 females, median age 28 years, range 18–52 years) referred to our institution for failure of primary ACL repair with DIS underwent surgery. In four patients, a single-stage ACLR was performed following the removal of the tibial monoblock. In six patients, due to arthrofibrosis and excessive tibial tunnel enlargement following the removal of the monoblock, arthroscopic arthrolysis and tunnel bone grafting were performed as a first-stage revision procedure. Patient characteristics and preoperative radiographic measurements are reported in Table 1. A brief description of each case is given below.

**Table 1:**
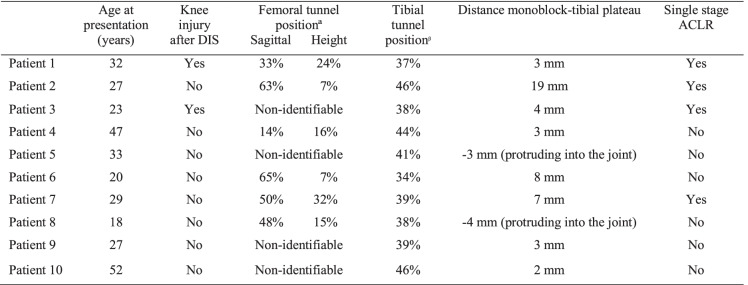
Patient characteristics and preoperative radiographic measurements

*ACLR* Anterior cruciate ligament reconstruction, *DIS* Dynamic Intraligamentary Stabilization

ªBernard and Hertel grid method

^β^Staubli and Rauschning method

### Patient 1

**Fig. 1:**
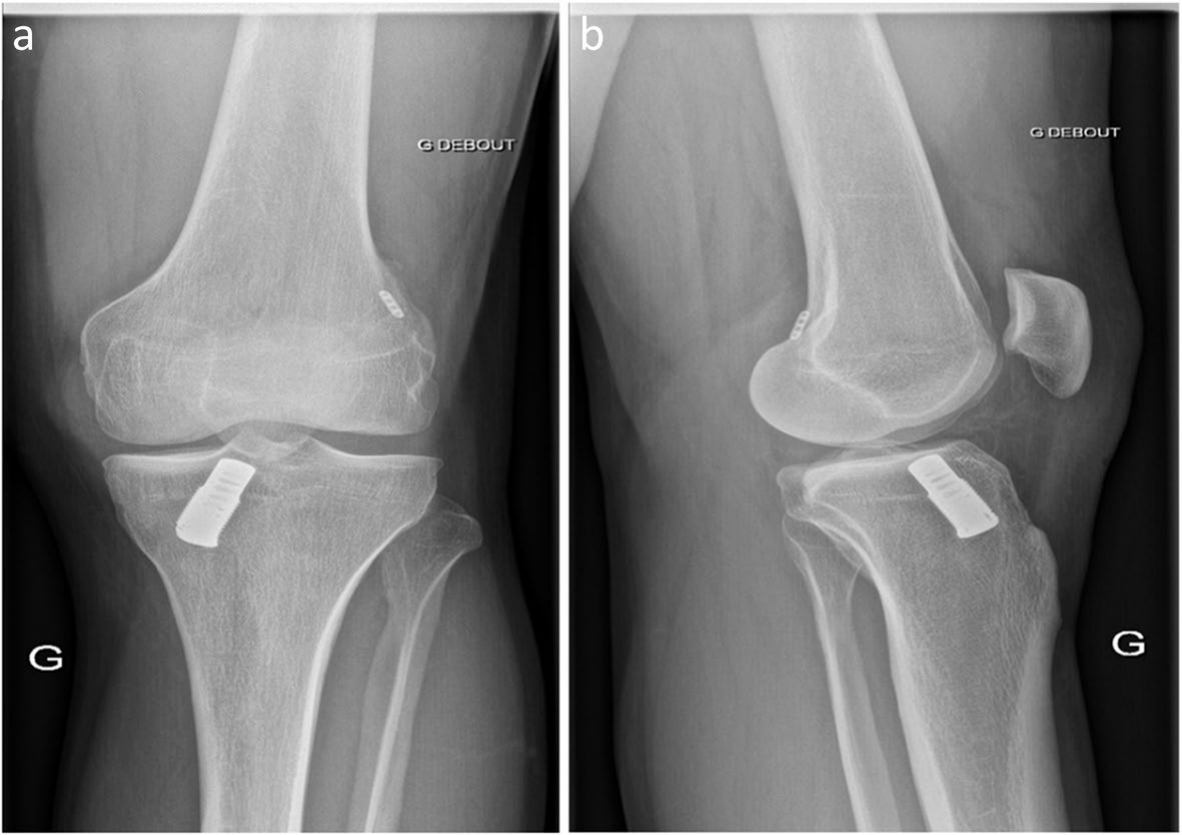
Preoperative anteroposterior **(a)** and lateral **(b)** X-rays before implant removal

A 32-year-old male twisted his knee while playing rugby about six months prior to presentation. Four years before, he underwent ACL repair with DIS. At the clinical examination, he had full range of motion (ROM) and positive Lachman and pivot-shift tests. The patient wished to return to his previous sport (rugby) and ACLR was therefore recommended. Preoperative anteroposterior and lateral X-rays showed the correct position of the femoral and tibial tunnels (Fig. 1). He underwent single-stage ACLR with a quadriceps tendon-bone autograft and medial meniscus resection. Diffuse synovitis was found during arthroscopic examination. Due to the large (11 mm) tibial bone defect following the removal of the monoblock, the tendinous part of the graft was inserted in the femoral tunnel and the bone plug was inserted in the tibial tunnel. The graft was fixed on both sides with bioabsorbable interference screws.

### Patient 2

A 27-year-old male presented with persistent knee pain and activity limitation about 10 months after he underwent ACL repair with DIS. He had a five-degree extension deficit, a negative Lachman test but a positive pivot-shift test. Due to activity limitations and a wish to return to football, ACLR was recommended. Arthroscopy revealed femoral tunnel malposition (anterior placement). Arthroscopic arthrolysis was performed and the tibial monoblock was removed. The patient underwent single-stage anatomical ACLR with a quadriceps tendon-bone autograft and lateral meniscus repair.

### Patient 3

A 23-year-old male presented with effusion, pain and instability following a knee injury that occurred a few days before while playing football. He underwent ACL repair with DIS 1.5 years before presentation. At the clinical examination, he had positive Lachman and pivot-shift tests. The tibial monoblock was removed and the patient underwent single-stage bone-patellar-tendon-bone autograft ACLR and repair of a medial meniscus ramp lesion.

### Patient 4

**Fig. 2:**
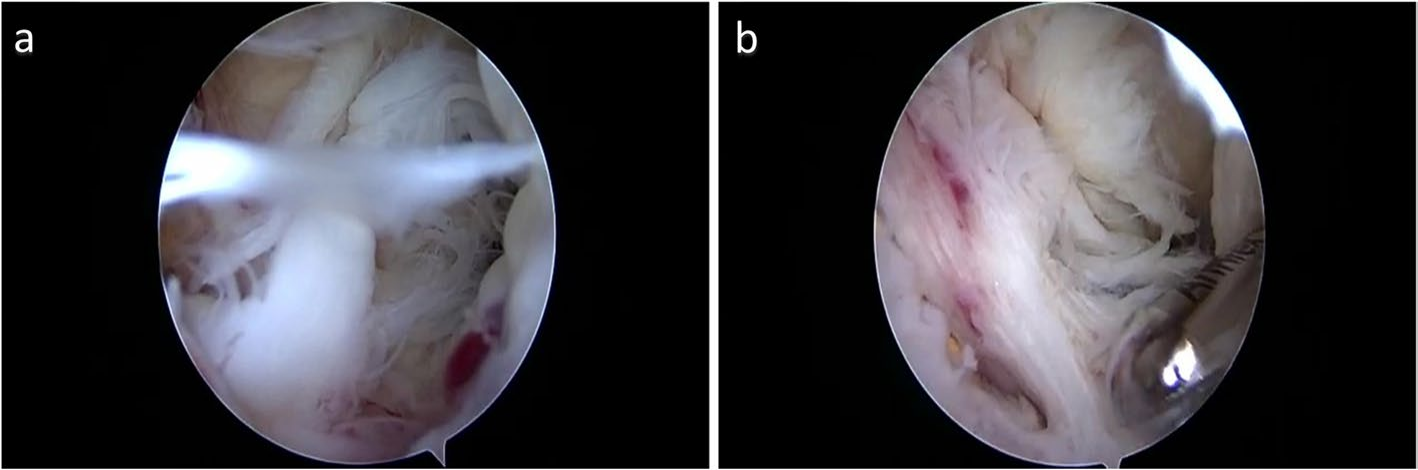
**a**-**b** Intraoperative arthroscopic pictures showing arthrofibrosis in the anterior compartment

A 47-year-old female who injured her ACL and underwent ACL repair with DIS one year before presentation. She complained of knee pain and instability. At the clinical examination, she had a severe (30 degrees) extension deficit and a positive Lachman test. The patient underwent arthroscopic arthrolysis due to generalised synovitis and arthrofibrosis (Fig. 2 a-b) and the removal of the tibial monoblock. The large tibial bone defect, which measured 15 mm in diameter, was bone grafted with a femoral head allograft. At the end of the procedure, the patient had an ROM of 0–140 degrees. A computed tomography scan performed four months after surgery showed the osteointegration of the bone graft within the tibial tunnel. She was scheduled for an ACLR in a second stage, but, due to other medical issues, she did not undergo surgery.

### Patient 5

A 33-year-old female who underwent ACL repair with DIS six months before and presented with knee pain and functional impairment. She had severely restricted ROM (10–60 degrees) and swelling. A synovial fluid examination showed no sign of infection. The patient underwent arthroscopic arthrolysis due to pronounced arthrofibrosis and removal of the tibial monoblock. The large tibial bone defect (12 mm diameter) was bone grafted with a femoral head allograft. The patient had an ROM of 0–140 degrees at the end of the procedure. She complained of persistent instability at the follow-up appointments (4, 7 and 12 months postoperatively) and an ACLR was recommended. However, the patient moved abroad and continued her treatment elsewhere.

### Patient 6

**Fig. 3:**
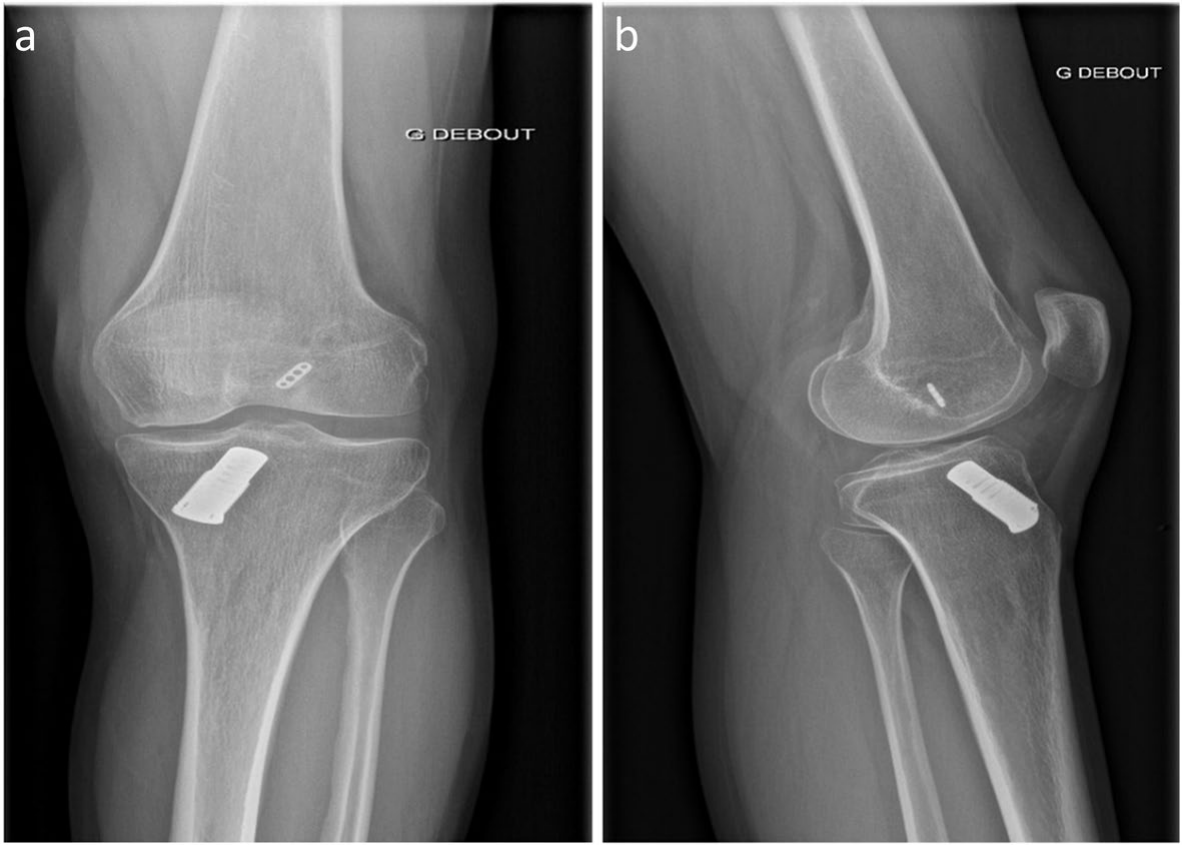
Preoperative anteroposterior **(a)** and lateral **(b)** X-rays before implant removal showing significant malposition of the femoral tunnel and improper position of the endobutton which had not flipped over the femoral cortex

A 20-year-old female presented with knee pain and significant functional impairment. She underwent ACL repair with DIS seven months before presentation. She had knee effusion, significant muscle atrophy and restricted ROM (10–100 degrees). Preoperative X-rays showed a significant malposition of the femoral tunnel. Moreover, the endobutton had not flipped over the femoral cortex (Fig. 3). The patient underwent two-stage ACLR. An arthroscopic arthrolysis due to generalised synovitis and arthrofibrosis and the removal of the tibial monoblock was performed in the first stage. Arthroscopy confirmed femoral tunnel malposition. The large tibial bone defect, which measured 15 mm in diameter, was bone grafted with a femoral head allograft. At the end of the procedure, the patient had an ROM of 0–140 degrees. An ACLR with hamstring tendons was performed seven months later, following the complete osteointegration of the bone allograft.

### Patient 7

A 29-year-old female presented with knee pain and instability. She underwent ACL repair with DIS 2.5 years before presentation. She had an ROM of 0–90 degrees and a positive Lachman test. During arthroscopic examination, scar tissue formation in the intercondylar notch was found and debrided. Following the removal of the tibial monoblock, the patient underwent single-stage quadriceps tendon-bone autograft ACLR. A concomitant lateral meniscus root repair and medial meniscus ramp lesion repair were performed.

### Patient 8

**Fig. 4:**
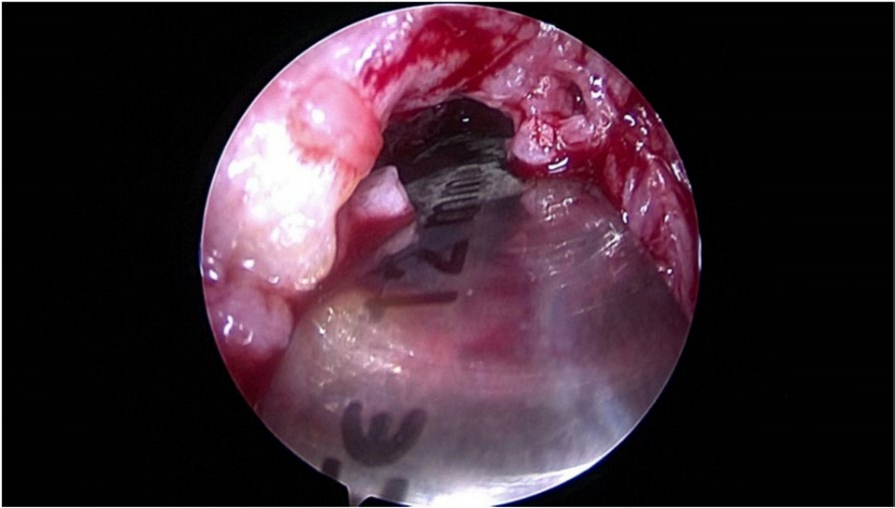
Intraoperative picture showing measurement of the enlarged (12 mm diameter) tibial tunnel after removal of the monoblock

An 18-year-old female presented with knee pain and swelling. She underwent ACL repair with DIS about four months before presentation and had an ROM of 15–80 degrees. Arthroscopy revealed a rupture of the ACL and generalised arthrofibrosis. The femoral tunnel was located in a non-anatomic, anterior position. The removal of the tibial monoblock left a large (12 mm diameter) bone defect (Fig. 4), which was bone grafted with a femoral head allograft. The patient is under follow-up to evaluate ACLR in a second stage.

### Patient 9

A 27-year-old female who underwent ACL repair with DIS prior to presentation. She had knee pain, a positive Lachman test and an ROM of 0–50 degrees. At arthroscopy, the ACL was partially torn and generalised arthrofibrosis was present. Extensive arthrolysis was performed. The removal of the tibial monoblock left a bone defect of 10–11 mm, which was bone grafted with a femoral head allograft. The patient was followed up for about four years. At the latest follow-up, she complained of subjective instability and had positive Lachman and pivot-shift tests. An ACLR was recommended, but the patient refused treatment due to her inability to pause her work as a cleaner.

### Patient 10

A 52-year-old female presented with pain in her knee such that normal walking was significantly limited. She underwent ACL repair with DIS about four months prior to presentation. Her ROM was 30–80 degrees. Arthroscopic arthrolysis due to generalised arthrofibrosis was performed. Arthroscopy also revealed a cyclops lesion, which was debrided together with the ACL remnant and the polyethylene wire. The removal of the tibial monoblock left a bone defect of 12 mm, which was bone grafted with a femoral head allograft. At the end of the procedure, the patient achieved an ROM of 0–110 degrees. She is under follow-up to evaluate a possible ACLR in a second stage.

## Discussion

The most important finding in this case series was that single-stage ACLR was performed in only four (40%) of ten patients following failure of ACL repair with DIS. Arthrofibrosis and excessive tibial tunnel enlargement following the removal of the monoblock prevented single-stage ACLR in six (60%) patients. These patients underwent arthroscopic arthrolysis and tibial tunnel bone grafting as a first-stage revision procedure.

DIS has been proposed as a technique for ACL repair to overcome the issue of cyclic loading of the repaired ligament during the healing phase [4]. The developers also suggested the addition of Steadman’s microfracturing technique [18] to further improve the healing response of the ACL. However, independent research groups have reported a high risk of complications such as re-tears/non-healing, arthrofibrosis and re-operations [13, 14], as well as a relatively low overall survival rate [15]. Senftl et al. [19] evaluated 105 patients who underwent DIS at a median follow-up of 21 months. The authors reported that 16.3% of the patients had insufficient functional stability and required subsequent ACLR. Younger age (< 24 years), a Tegner score of more than 5 and postoperative side-to-side laxity of > 2 mm have been associated with an increased likelihood of ACL revision surgery after DIS [20]. Despite the reported high complication and failure rate with this technique, there is a lack of literature evaluating the potential challenges of ACLR following failure of DIS.

Knee arthrofibrosis is a known complication after ACL repair with DIS [13, 14, 21] and was a factor which prevented single-stage ACLR in the present case series. Häberli et al. [21] hypothesised that the reduced range of motion could be due to excessive scar tissue formation at the ACL repair site. Ateschrang et al. [22] performed a diagnostic arthroscopy six months after DIS and found that scar tissue formation in the notch was common, which the authors attributed to microfracture of the ACL femoral footprint. Another potential reason related to arthrofibrosis with DIS might be the polyethylene wire which is left within the joint. Other ACL repair techniques [6, 7, 23] did not report problems such as restricted range of motion or arthrofibrosis. However, in the present case series, arthrofibrosis and restricted range of motion might be also related to technical errors. Some of the patients presented with femoral tunnel malposition and/or monoblock malposition (protruding into the joint), which might have contributed to the development of these complications. Moreover, information about preoperative and postoperative rehabilitation after ACL repair was not available. This might also have potentially affected the development of knee arthrofibrosis.

The large tibial bone defect following the removal of the monoblock was the other factor which prevented single-stage ACLR in the present case series. The bulky monoblock used for DIS might be a major downside of this ACL repair technique. According to the developers [21], one of the main advantages of ACL repair with DIS is that, in the event of revision surgery, ACLR would be performed as a single-stage procedure with no need for bone grafting. However, in many (60%) cases in the present series, the bone defect left following the removal of the monoblock was too large to perform a single-stage ACLR and required bone grafting. The diameter of the tibial tunnel usually became larger than the 10 mm monoblock after debridement of the fibrotic tissue which developed at the metal-bone interface. It might be argued that a possible option to reduce the future risk of two-stage ACLR would be to remove the bulky tibial monoblock and bone graft the tibial tunnel routinely after ACL repair with DIS. This hardware stabilises the knee, reducing anterior tibial translation only temporarily during ACL healing [20]. Moreover, previous studies have reported that the hardware is removed anyway in a significant (40-62.3%) number of patients due to local discomfort [19, 20, 24].

This study is important as it is, to the authors’ knowledge, the first report describing the surgical challenges following failure of ACL repair with DIS. Patients should be informed that there is a high risk of two-stage ACLR in the event of failure of ACL repair with DIS. This is because of the arthrofibrosis and/or the large tibial bone defect resulting from the removal of the monoblock.

The main limitation of this study is the small number of patients. ACL repair with DIS is a technique that is rarely used in our geographical area. All the patients included in this series underwent ACL repair with DIS elsewhere and were referred to our institution because of postoperative problems or new trauma. Some information about the primary surgery (location of ACL injury, time from injury to surgery, preoperative ROM/effusion, pre- and postoperative rehabilitation) was not available. However, the aim of this study was not to describe the results or complications of ACL repair with DIS but instead to evaluate the surgical challenges in the event of revision of a failed DIS. Finally, this case series represents a single-centre and single-surgeon experience. It might be argued that the decision not to perform a single-stage ACLR was dependent on the operating surgeon and was then potentially biased. However, this decision was based on well-known principles of ACL surgery. Full knee extension should be achieved before ACLR [25, 26] and bone grafting is recommended with a tunnel diameter of 10–15 mm [27].

## Conclusion

In the present case series, single-stage ACLR was performed in only four (40%) of ten patients following failure of ACL repair with DIS. Arthrofibrosis and excessive tibial tunnel enlargement following the removal of the monoblock prevented single-stage ACLR in six (60%) patients. It is important for clinicians to inform patients that, in the event of failure of ACL repair with DIS, they may run a high risk of undergoing two-stage ACLR.

## References

[1] Diermeier T, Rothrauff BB, Engebretsen L, Lynch AD, Ayeni OR, Paterno MV, Xerogeanes JW, Fu FH, Karlsson J, Musahl V, Svantesson E, HamrinSenorski E, Rauer T, Meredith SJ (2020). Panther symposium ACL treatment consensus group. treatment after anterior cruciate ligament injury: panther symposium ACL treatment consensus group. Knee Surg Sports Traumatol Arthrosc..

[2] Nwachukwu BU, Patel BH, Lu Y, Allen AA, Williams RJ (2019). Anterior cruciate ligament repair outcomes: an updated systematic review of recent literature. Arthroscopy.

[3] Achtnich A, Herbst E, Forkel P, Metzlaff S, Sprenker F, Imhoff AB, Petersen W (2016). Acute proximal anterior cruciate ligament tears: outcomes after arthroscopic suture anchor repair versus anatomic single-bundle reconstruction. Arthroscopy.

[4] Kohl S, Evangelopoulos DS, Ahmad SS, Kohlhof H, Herrmann G, Bonel H, Eggli S (2014). A novel technique, dynamic intraligamentary stabilization creates optimal conditions for primary ACL healing: a preliminary biomechanical study. Knee.

[5] Schliemann B, Glasbrenner J, Rosenbaum D, Lammers K, Herbort M, Domnick C, Raschke MJ, Kösters C (2018). Changes in gait pattern and early functional results after ACL repair are comparable to those of ACL reconstruction. Knee Surg Sports Traumatol Arthrosc.

[6] Listvan der JP, DiFelice GS (2017). Range of motion and complications following primary repair versus reconstruction of the anterior cruciate ligament. Knee.

[7] Eckvan CF, Limpisvasti O, ElAttrache NS (2018). Is there a role for internal bracing and repair of the anterior cruciate ligament? A systematic literature review. Am J Sports Med.

[8] Ahmad SS, Schreiner AJ, Hirschmann MT, Schröter S, Döbele S, Ahrend MD, Stöckle U, Ateschrang A (2019). Dynamic intraligamentary stabilization for ACL repair: a systematic review. Knee Surg Sports Traumatol Arthrosc.

[9] Eggli S, Kohlhof H, Zumstein M, Henle P, Hartel M, Evangelopoulos DS, Bonel H, Kohl S (2015). Dynamic intraligamentary stabilization: novel technique for preserving the ruptured ACL. Knee Surg Sports Traumatol Arthrosc.

[10] Büchler L, Regli D, Evangelopoulos DS, Bieri K, Ahmad SS, Krismer A, Muller T, Kohl S (2016). Functional recovery following primary ACL repair with dynamic intraligamentary stabilization. Knee.

[11] Eggli S, Röder C, Perler G, Henle P (2016). Five year results of the first ten ACL patients treated with dynamic intraligamentary stabilization BMC Musculoskelet Disord.

[12] Henle P, Röder C, Perler G, Heitkemper S, Eggli S (2015). Dynamic Intraligamentary Stabilization (DIS) for treatment of acute anterior cruciate ligament ruptures: case series experience of the first three years. BMC Musculoskelet Disord.

[13] Meister M, Koch J, Amsler F, Arnold MP, Hirschmann MT (2018). ACL suturing using dynamic intraligamentary stabilisation showing good clinical outcome but a high reoperation rate: a retrospective independent study. Knee Surg Sports Traumatol Arthrosc.

[14] Osti M, El Attal R, Doskar W, Höck P, Smekal V (2019). High complication rate following dynamic intraligamentary stabilization for primary repair of the anterior cruciate ligament. Knee Surg Sports Traumatol Arthrosc.

[15] Ahmad SS, Schürholz K, Liechti EF, Hirschmann MT, Kohl S, Klenke FM (2020). Seventy percent long-term survival of the repaired ACL after dynamic intraligamentary stabilization. Knee Surg Sports Traumatol Arthrosc.

[16] Bernard M, Hertel P, Hornung H, Cierpinski T (1997). Femoral insertion of the ACL. Radiographic quadrant method. Am J Knee Surg..

[17] Stäubli HU, Rauschning W (1994). Tibial attachment area of the anterior cruciate ligament in the extended knee position. Anatomy and cryosections in vitro complemented by magnetic resonance arthrography in vivo. Knee Surg Sports Traumatol Arthrosc..

[18] Steadman JR, Cameron-Donaldson ML, Briggs KK, Rodkey WG (2006). A minimally invasive technique ("healing response") to treat proximal ACL injuries in skeletally immature athletes. J Knee Surg.

[19] Senftl M, Petek D, Jacobi M, Schallberger A, Spycher J, Stock A, Hess R, Tannast M (2021) Occurrence of inadequate ACL healing after Dynamic Intraligamentary Stabilization and functional outcome-a multicentre case series. Eur J Orthop Surg Traumatol. 10.1007/s00590-021-03096-9. Epub ahead of print. PMID: 34430988. 10.1007/s00590-021-03096-9 PMC9433353 34430988

[20] Henle P, Bieri KS, Brand M, Aghayev E, Bettfuehr J, Haeberli J, Kess M, Eggli S (2018). Patient and surgical characteristics that affect revision risk in dynamic intraligamentary stabilization of the anterior cruciate ligament. Knee Surg Sports Traumatol Arthrosc.

[21] Häberli J, Jaberg L, Bieri K, Eggli S, Henle P (2018). Reinterventions after dynamic intraligamentary stabilization in primary anterior cruciate ligament repair. Knee.

[22] Ateschrang A, Ahmad SS, Stöckle U, Schroeter S, Schenk W, Ahrend MD (2018). Recovery of ACL function after dynamic intraligamentary stabilization is resultant to restoration of ACL integrity and scar tissue formation. Knee Surg Sports Traumatol Arthrosc..

[23] DiFelice GS, van der List JP (2018). clinical outcomes of arthroscopic primary repair of proximal anterior cruciate ligament tears are maintained at mid-term follow-up. Arthroscopy.

[24] Kohl S, Evangelopoulos DS, Schär MO, Bieri K, Müller T, Ahmad SS (2016). Dynamic intraligamentary stabilisation: initial experience with treatment of acute ACL ruptures. Bone Joint J..

[25] Gage A, Kluczynski MA, Bisson LJ, Marzo JM (2019). Factors associated with a delay in achieving full knee extension before anterior cruciate ligament reconstruction. Orthop J Sports Med..

[26] Shelbourne KD, Patel DV (1995). Timing of surgery in anterior cruciate ligament-injured knees. Knee Surg Sports Traumatol Arthrosc.

[27] Salem HS, Axibal DP, Wolcott ML, Vidal AF, McCarty EC, Bravman JT, Frank RM (2020). Two-stage revision anterior cruciate ligament reconstruction: a systematic review of bone graft options for tunnel augmentation. Am J Sports Med.

